# An Ancient Skeleton with Multiple Osteoblastic Bone Lesions Containing a Scapular Sunburst Appearance from a 5th–6th Century Grave Excavated in Oita, Japan

**DOI:** 10.1155/2018/1659510

**Published:** 2018-09-06

**Authors:** Toshiyuki Tsurumoto, Tetsuaki Wakebe, Keiko Ogami-Takamura, Keishi Okamoto, Kazunori Tashiro, Kazunobu Saiki

**Affiliations:** ^1^Department of Macroscopic Anatomy, Graduate School of Biomedical Science, Nagasaki University, Japan; ^2^Nagasaki Medical College, Japan; ^3^Tashiro Clinic, Tara Town, Saga Prefecture, Japan

## Abstract

A human skeleton of a middle-aged adult male was found in a 5th–6th century Kinoue-Kodo stone coffin excavated from the southwest marginal region of the Oita plains, northeast Kyushu, Japan. The skeleton was buried respectfully in the ancient tomb, and red pigment was applied to his face after death. We report herein findings from computed tomography imaging of the skeleton and discuss the multiple osteoblastic lesions identified in the humerus, scapula, clavicle, vertebra, pelvic bones, and skull of this individual. These lesions comprised cortical bone thickening with periosteal reaction localized to the surface and osteosclerotic changes mainly observed in the trabecular structure of cancellous bone. In particular, a typical sunburst pattern was also noted on the left scapula as another characteristic lesion found in this case. By differential diagnosis, the disease suffered by this individual was most likely to be metastatic bone tumors, especially of prostate cancer. This person may have survived until many bone metastases had developed throughout his whole body.

## 1. Introduction

In archaic societies in which the natural and social environment surrounding human living areas and the demographic composition were markedly different from the present time, it might be thought that the incidence of cancer was lower than nowadays. However, the frequency of malignant tumors developing in young people (adolescents and young adults), such as osteosarcoma and leukemia, may not have been so different. When ancient human skeletons excavated from ruins are observed, benign tumors, such as osteomas of various sizes, are not rare, but it is very rare to discover traces of malignant tumors in a skeleton. In this study, we closely investigated multiple bone lesions noted in an adult male human skeleton buried in a 5th–6th century sarcophagus tomb of the Kofun period found in the plains of Oita prefecture, Kyushu, Japan.

## 2. Materials and Methods

**Figure 1:**
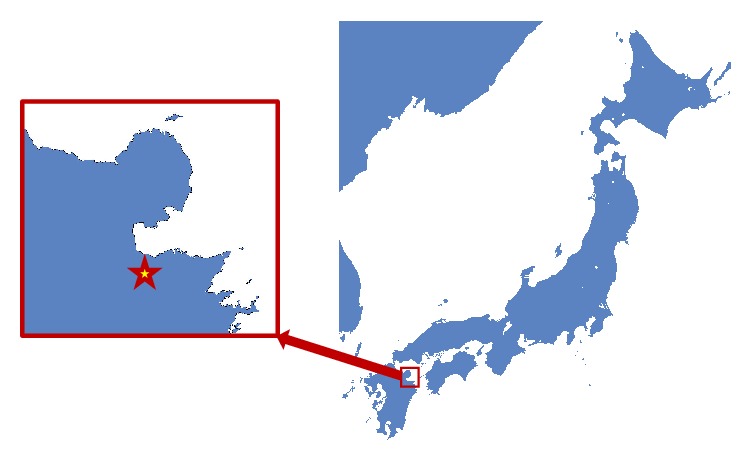
Remains of the Kinoue-Touge stone coffin group located in Oita city, Oita Prefecture, Japan.

**Figure 2:**
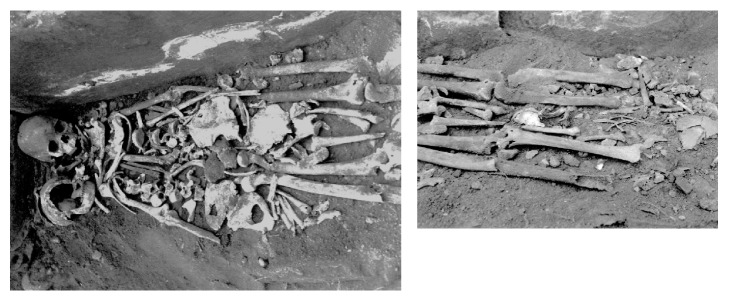
Photographs of two adult human skeletons buried in one of the Kinoue-Kodo stone coffins. The subject was the human skeleton on the left, which was considered to be a middle-aged male.

This human skeleton was excavated from a Kinoue-Kodo stone coffin in a Kinoue-Touge stone coffin group located in the southwest marginal region of the Oita plains of northeast Kyushu (Figure 1). According to the archeological findings, this ruin was considered to have been constructed in the 5th–6th century. There was a big ancient tomb in this region that was an “ancient imperial tomb” of 80 m in length, 58 m in diameter, and 9 m in height. It is considered to be the tomb of a powerful family ruling this region in the middle of the 5th century. Kinoue-Kodo stone coffins were discovered near this imperial ancient tomb, and three box-shaped stone coffins constructed by combining plate-like broken stones with an andesite nature were aligned in a south–north direction. At least two adult human skeletons were buried in one of these coffins. We report here on the human skeleton on the left, which was considered to be a middle-aged adult male (Figure 2). In previous report, Tashiro [1] speculated that this human skeleton in a ‘Paleopathological study of skeletons excavated from Kyushu Island', in which this individual was considered to have prostate cancer before death. We report this case in detail with computed tomography (CT) images and discuss the case in relation to the current literatures. The CT images of the pathological bones in this skeleton were obtained by using clinical CT equipment (Activision 16, Toshiba Corp. Japan); the sampling interval was 0.5 mm, and the data were saved in DICOM (Digital Imaging and Communication in Medicine) format. Free DICOM Viewer 1.4.5.0 (YAKAMI Software) software was used to display them.

## 3. Result

### 3.1. Condition of the Human Skeleton

**Figure 3:**
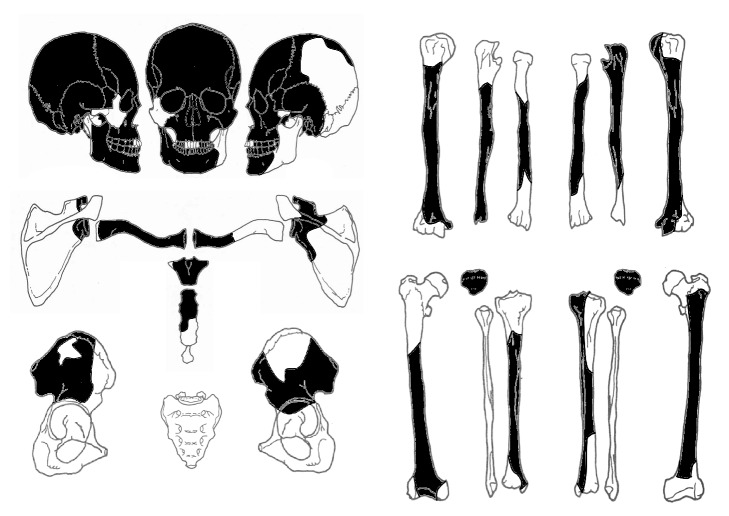
Condition of the human skeleton. The skull, bilateral humeri, ulnas, femora, tibiae, clavicles, scapulae, sternum, patella, and pelvic bones remained mostly or partially.

As shown in Figure 3, the skull, bilateral humeri, ulnas, radii, femora, tibiae, clavicles, scapulae, sternum, and pelvic bones were mostly or partially present. The condition of the remaining skull was favorable, and red pigment had been applied to the surface, mainly on the frontal face, after death.

### 3.2. Skull (Figure 4)

**Figure 4:**
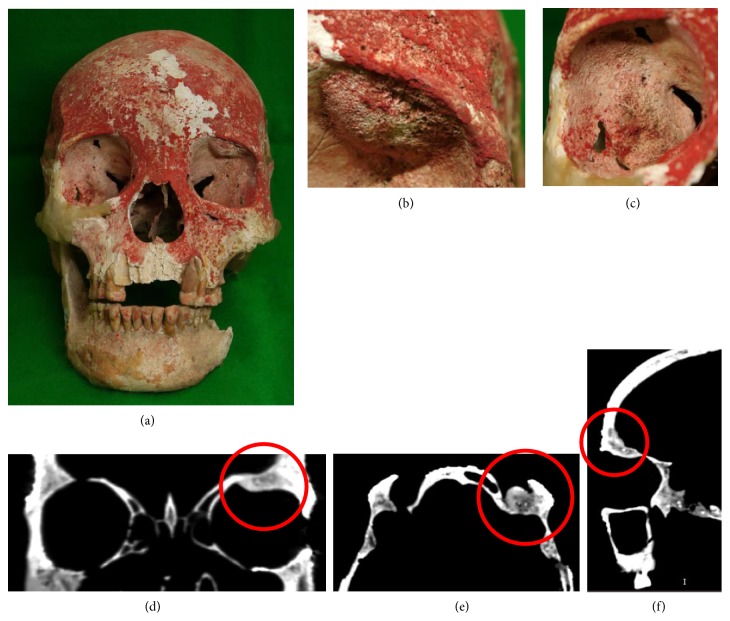
Photographs of the skull. Red pigment was applied mainly on the frontal face region after death (a). In the left superior orbital wall, a 15 × 15 mm bulging lesion with a slightly porous surface was present (b) in which a solid osteoblastic lesion continuous with the inner region was noted on CT images (d, e, and f). On the inner wall surface of the right orbit, a 20 × 20 mm irregular prominent lesion was present (c).

The skull mostly remained in a relatively favorable condition excluding the right zygomatic bone, most of the left parietal bone, and part of the occipital bone. It was also clear that red pigment had been placed on the surface, mainly on the frontal face, after death, and the superficial layer of the bone surface had come off to various extents (Figure 4(a)). Thus, macroscopic observation of some parts was difficult. First, the appearance was observed. The metopic suture remained over the entire length in the midline of the frontal bone. Bulging lesions were present on the cortical bone surface of the bilateral inferior orbital walls. A 15 × 15 mm bulging lesion with a slightly porous surface was present on the left superior orbital wall (Figure 4(b)). On CT images, a solid osteoblastic lesion continuing between the inner and outer cortex was noted (Figures 4(d), 4(e), and 4(f)). In addition, a 20 × 20 mm prominent irregular lesion was present on the outer wall surface of the right orbit, in which a spicular formation showing periosteal reaction was noted on the surface (Figure 4(c)). At present, macroscopic observation of the inner surface of the skull is not possible.

### 3.3. Left Scapula (Figure 5)

**Figure 5:**
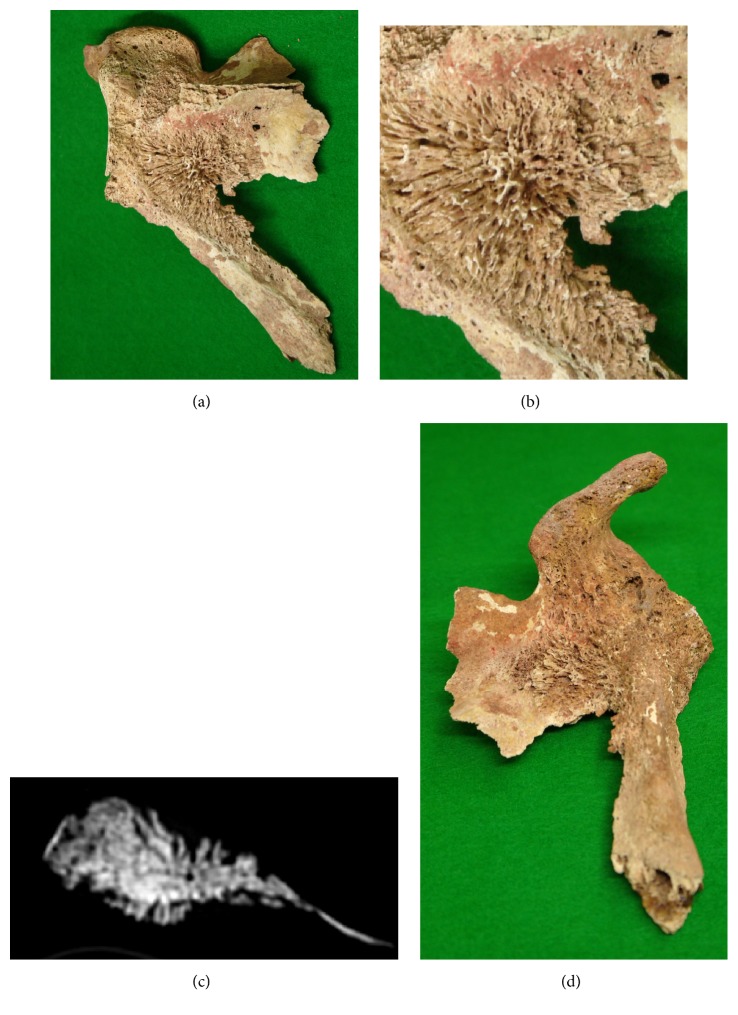
Photographs of the sunburst appearance noted on the left scapula. An osteoblastic lesion was preset, comprised of small, densely concentrating, needle-like or plate-like protruding bones expanding in a 35 × 40 mm posterior region at the base of the glenoid cavity below the base of the scapular spine (a, b). This lesion continued to the proliferative lesion on the bone surface located at the base of the coracoid (d). On CT images, the presence of needle-like osteoblastic lesions was confirmed expanding continuously across both the anterior and posterior surfaces of the base of the glenoid cavity (c).

The left scapulae, mainly the glenoid cavity region, remained partially. Of its glenoid cavity, the coracoid, lateral margin, and base of the scapular spine were continuous and remained as one bone fragment. The first finding that attracted attention was an osteoblastic lesion comprised of densely concentrating small needle-like or plate-like protruding bone expanding over in a 35 × 40 mm region on the posterior surface of the base of the glenoid cavity below the base of the scapular spine (Figures 5(a) and 5(b)). The angle changed in a regular manner from the central area to the marginal region, and its overall morphology showed a sunburst appearance. The lesion was continuous with proliferative lesions on the bone surface at the base of the coracoid. In addition, a lesion with a sunburst appearance, although it was incomplete, was also present on the anterior surface of the left scapula, mainly at the base of the coracoid (Figure 5(d)). In CT images of the left scapula, the presence of needle-like osteoblastic lesions was confirmed expanding continuously on both the anterior and posterior surfaces at the base of the glenoid cavity (Figure 5(c)).

### 3.4. Right Scapula, Right Clavicle, and Vertebral Body (Figure 6)

**Figure 6:**
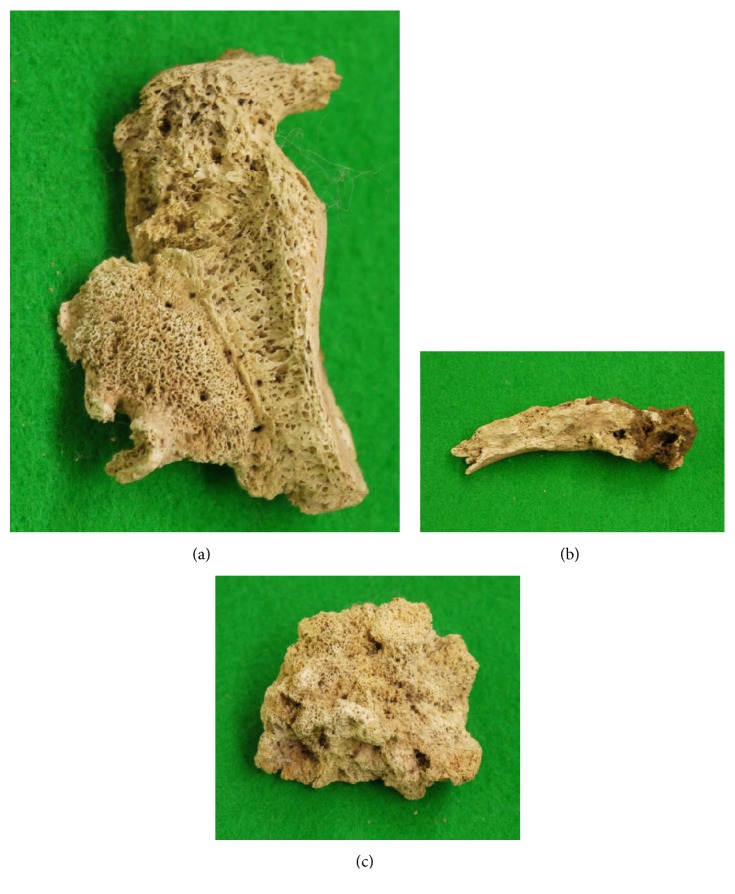
Photographs of the right scapula (a), right clavicle (b), and vertebral body (c). The right scapula, mainly the glenoid region, remained partially (a). Overall, the bone was markedly broken, but osteoblastic lesions were present extensively on the bone surface excluding the joint surface. On the right clavicle, worm eaten-like lesions were present almost circumferentially on the surface (b). On the vertebral body (c), cancellous bone was markedly thickened across its entirely, showing the features of osteosclerosis.

The right scapula, mainly the glenoid cavity region, remained partially (Figure 6(a)). Overall, the bone was markedly broken, but osteoblastic lesions were present extensively on the bone surface excluding the joint surface.

About one-third of the proximal region of the right clavicle remained, but this was also broken (Figure 6(b)). However, on macroscopic observation of the surface, worm eaten-like lesions were present almost circumferentially. On CT images, intermixed osteosclerosis and osteolysis were continuous from the bone surface to the inner region.

One vertebra remained, but the condition was poor and only the vertebral body remained partially (Figure 6(c)). Based on the size, it was considered to be a thoracic or lumbar vertebra, but it was unclear which vertebra it was. Cancellous bone was markedly thickened over the entire residual vertebral body, appearing to be osteosclerosis. This was also noted on CT images.

### 3.5. Right Humerus (Figure 7)

**Figure 7:**
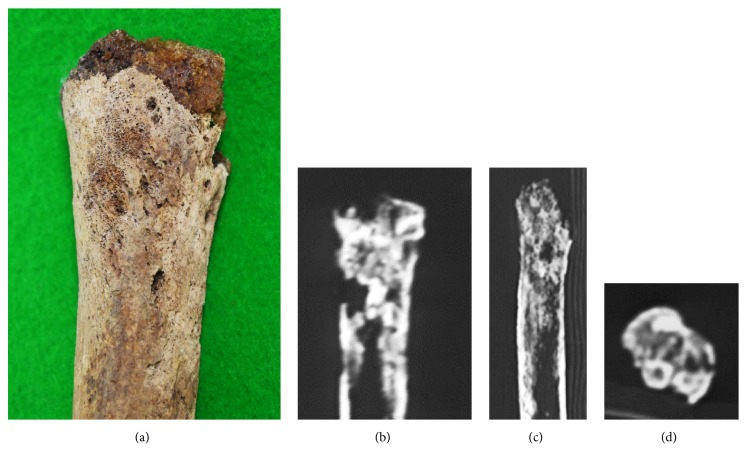
Photographs of the right humerus. Lesions showing irregular minute osteoblastic changes were observed on the anterior surface of the proximal region and bone surface of the crest of the lesser tubercle over the intertubercular sulcus (a). On CT, continuous osteosclerosis was observed from the superficial layer to the inner region in the proximal metaphysis of this humerus (b-d).

In the right humerus, the proximal end containing the entire bone head and distal joint surface was missing. Of the remaining regions, lesions showing irregular minute osteoblastic changes were present on the anterior surface of the proximal region and on the bone surface of the lesser tubercle over the intertubercular sulcus (Figure 7(a)). On CT images, osteosclerosis was observed continuing from the superficial layer to the inner region and in the proximal metaphysis of this humerus (Figures 7(b), 7(c), and 7(d)).

### 3.6. Pelvic Bones (Figure 8)

**Figure 8:**
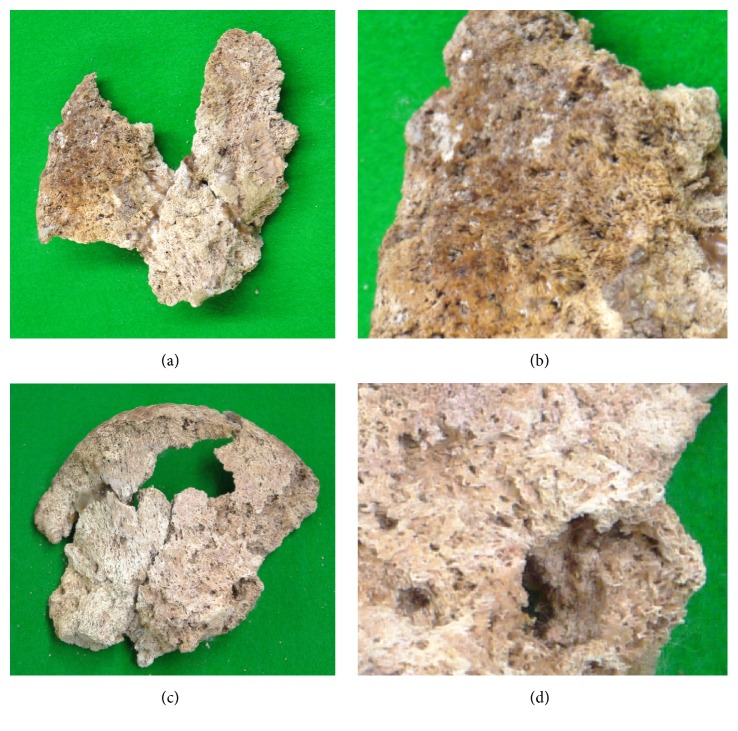
Photographs of the bilateral pelvic bones. The condition was poor on the right side (a, b) and left side (c, d). In the remaining region, either cortical or cancellous bone was replaced with osteoblastic lesions almost entirely from the surface to the inner region.

The pelvic bones remained as a partial block centered on the ilium on both sides. The right coxa was present as a block containing the anterior half of the ala of the ilium and part of the acetabular roof (Figures 8(a) and 8(b)). The left coxa remained as a bone fragment containing the anterior part of the auricular surface of the ilium (Figures 8(c) and 8(d)). Because both coxae were predominantly broken, accurate macroscopic evaluation of the properties was difficult, but cortical and cancellous bones were replaced with osteoblastic lesions from the surface over the inner region across most of the remaining region; i.e., the surface was rough and irregular, showing a periosteal osteoblastic pattern, and it did not stay on the bone surface but instead continued into the deep inner region. The trabecula was markedly thickened in partially glimpsed cancellous bone. These lesions had properties similar to changes in the vertebral body described above. In CT images, osteosclerosis and osteolysis continuing from the bone surface to the inner region were mixed, similar to that noted in the vertebral body and clavicle.

In addition, the left humerus and bilateral ulnas, femora, tibiae, and patellae remained partially, but no lesions were noted on the surfaces of any bone from macroscopic observations.

## 4. Discussion

### 4.1. Diseases to Be Subjected to Differential Diagnosis

The characteristics of bone lesions in this case included the presence of multiple osteoblastic lesions throughout bones from across the entire body, and these were comprised of cortical bone thickening with periosteal reaction localized to the surface and osteosclerosis mainly showing changes in the trabecular structure of cancellous bone. Based on these findings, the diseases present in this case were differentiated as described below.

A typical disease responsible for the development of osteoblastic bone tumors could be a representative primary malignant bone tumor, such as osteosarcoma. Smith-Guzman [2] reported a case of primary malignant bone tumor observed in the right humerus excavated from a Pre*-*Columbian era ruin from A.D. 1265–1380, Aufderheide [3] reported a case of osteosarcoma showing a typical sunburst appearance, which developed in the distal humerus and femur in an adult native Peruvian 800 years ago, and Bona [4] reported the results of an investigation of osteosarcoma in the right humerus using a biomarker in a 25–35-year-old female excavated from an approximately 2,000-year-old ruin. All these cases developed in a solitary bone. Osteosarcoma is considered to be a highly malignant disease, and it metastasizes to visceral organs from an early stage, but the development of multiple bone lesions in distant regions is unusual. Accordingly, osteosarcoma is unlikely to be the disease in the present ancient case without any available therapy.

Another representative primary malignant bone tumor, Ewing sarcoma, shows osteolysis on radiography as a rule, such as a permeative (small holes) or moth-eaten (mottled) appearance, but these may be accompanied by periosteal reaction, expressed as lamellated, sunburst, or spiculated patterns, in proximity, and it may show Codman's triangle similar to osteosarcoma. In cases intensively treated using modern medical care, the development of several bone lesions is not rare. Therefore, although the possibility is low, it cannot be ruled out that this was a case of Ewing sarcoma.

Among metastatic bone tumors, diseases showing an osteoblastic pattern are candidates for differential diagnosis. Klaus [5] observed osteoblastic and osteolytic lesions in the lumbar spine and sacrum in four human skeletons excavated from ancient ruins in Peru from the period A.D. 900–1600 and considered that these were cases with metastatic lesions of prostate cancer. They considered blood diseases, such as myeloma, leukemia, and Hodgkin's and non-Hodgkin's lymphomas, bone- and cartilage-derived primary malignant tumors, such as sclerosing osteosarcomas and chondrosarcomas, and benign and malignant tumors, such as meningiomas, hemangiomas, and adrenal neuroblastomas, for differential diagnosis in the diagnostic course. It was considered that bone metastasis of prostate cancer was the most suspicious for the development of multiple osteoblastic lesions in bones throughout the whole body.

Furthermore, to infer the diagnosis of the present case, the characteristic periosteal proliferation, i.e., the sunburst pattern, observed on the dorsal surface of the left scapula may be the key. Periosteal reaction on the cortical bone surface occurs due to elevation of the bone surface periosteum caused by various bone tumors, infection, trauma, drug administration, or arthritis, and it shows various patterns on radiography depending on the intensity, aggressiveness, and duration of the underlying insult of the lesion [6]. Of these, a sunburst pattern is observed when rapidly growing tumors develop simultaneously in bone and the surrounding tissue without destructive changes [7]. Spiculated periosteal reaction is most frequently noted in primary bone tumors, but it is also sometimes observed in metastatic bone tumors.

Vilar [8] summarized six cases of metastatic bone tumor and stated that bone metastasis should be included in differential diagnosis of perpendicular periosteal reaction at 40-years-old or older. Lehrer [7] also reported five similar cases where the primary lesion was retinoblastomas in two cases, prostatic carcinoma in one case, tumor of undetermined primary origin (probably bronchogenic) in one case, and chloromatous acute myelocytic leukemia in one case. Bloom [9] surveyed 20 periosteal sunburst reaction cases of metastatic bone tumors, in which the most frequent primary lesion was prostate cancer. Therefore, periosteal reaction or sunburst periosteal proliferation may occur in metastatic bone tumors similar to that in primary bone tumors. In the present human skeleton, multiple osteoblastic lesions were present in bones throughout the whole body, but osteolytic lesions were almost absent in the remaining bones, and the skeleton was judged as a middle-aged male. Based on these findings, it was considered that metastatic bone tumors, such as those from prostate cancer, were the most likely lesion. Furthermore, Hove [10] initially reported two clinical cases in which spicule formation was noted in metastatic vertebral body lesions of prostate cancer on CT images.

### 4.2. Paleopathological Report on Cases of Bone Metastasis of Prostate Cancer

In paleopathological samples, several cases of bone metastasis of prostate cancer have been reported. Wakely [11] reported findings suggestive of metastatic lesions of prostate cancer observed using radiography and scanning electron microscopy in an elderly male skeleton excavated from a medieval ruin from the 14th century in the Canterbury region of England. Schultz [12] noted rough bone-forming lesions on the iliac and costal bone surfaces in a male human skeleton from 2,700 years ago in his 40–50s excavated from South Siberia and considered that it was a case of bone metastasis of prostate cancer. Ghabili [13] reviewed 17 cases of bone metastasis of prostate cancer, including bones excavated from approximately 3,300-year-old ruins in Spain and a case excavated from a tomb in England in 1834, in which the affected bones included the pelvis, femur, spine, clavicle, skull, scapula, humerus, ulna, radius, fibula, carpal, and tarsal bones, showing extensive distribution. More than half of these were an osteogenesis/osteolysis mixture, but five cases contained only osteoblastic lesions. Osteoblastic lesions were widely distributed in the axial skeleton, such as the pelvis, scapula, costal bone, and spine, and four extremity bones, such as the humerus and femur. Of these, the clavicle of a skeleton excavated from a 19th-century tomb reported by Waldron [14] was the only case showing a “sunburst pattern” similar to that in the present case.

## 5. Conclusion

This skeleton with multiple metastatic bone tumors was buried respectfully in an ancient tomb and red pigment was applied to the face after death. Among the Yayoi people in the Kofun period in Japan, vermilion color was applied to the face with mercury pigment and red iron oxide as a burial ritual. In this study, red pigment was also applied to the facial bone. Archeologically, it is considered that the red color was magical and sacred and had meaning as a “talisman” or hoping for ‘resurrection of the dead'. Considering these findings, this person appears to have been buried respectfully in an ancient tomb after death. As for this case, the most likely disease was suggested to be metastatic bone tumors, probably from prostate cancer. Among malignant tumors, prostate cancer shows relatively slow progress. Therefore, this person might have survived until many bone metastases developed throughout the whole body.

## Data Availability

The data used to support the findings of this study are included within the article.
